# Metagenomic Analyses Reveal Distinct Gut Microbiota Signature for Predicting the Neoadjuvant Chemotherapy Responsiveness in Breast Cancer Patients

**DOI:** 10.3389/fonc.2022.865121

**Published:** 2022-04-01

**Authors:** Yuanyuan Li, Bingbin Dong, Wei Wu, Jiawei Wang, Hao Jin, Kangmei Chen, Kangling Huang, Songyin Huang, Yandan Yao

**Affiliations:** ^1^ Breast Tumor Center, Sun Yat-sen Memorial Hospital, Sun Yat-sen University, Guangzhou, China; ^2^ Shenshan Medical Center, Sun Yat-sen Memorial Hospital, Sun Yat-sen University, Shanwei, China; ^3^ Guangdong Provincial Key Laboratory of Malignant Tumor Epigenetics and Gene Regulation, Sun Yat-sen Memorial Hospital, Sun Yat-sen University, Guangzhou, China; ^4^ Biotherapy Center, Sun Yat-Sen Memorial Hospital, Sun Yat-Sen University, Guangzhou, China

**Keywords:** gut microbiota, breast cancer, neoadjuvant chemotherapy, pathologic response, CD4^+^ T lymphocytes

## Abstract

**Background:**

Growing evidence supports the modulatory role of human gut microbiome on neoadjuvant chemotherapy (NAC) efficacy. However, the relationships among the gut microbiome, tumor-infiltrating lymphocytes (TILs), and NAC response for breast cancer (BC) patients remain unclear. We thus proposed this preliminary study to investigate the relationship between gut microbiome and BC patients’ responses to NAC treatment as well as underlying mechanisms.

**Methods:**

Prior to receiving NAC, the fecal metagenome collected from 23 patients with invasive BC was analyzed. Patients were subsequently assigned to the NAC non-effectual group and the NAC effectual group based on their response to NAC. The peripheral T lymphocyte subset counts were examined by flow cytometry methods. CellMinor analysis was employed to explore the relationship between CD4 mRNA expression and the reaction of tumor cells to NAC drugs.

**Results:**

The gut microbiomes of the NAC non-effectual group showed characteristics of low diversity with low abundances, distinct metagenomic composition with decreased butyrate-producing and indolepropionic acid-producing bacteria, and increased potential pathobionts compared with the NAC effectual group. The combination of *Coprococcus, Dorea*, and uncultured *Ruminococcus* sp. serves as signature bacteria for distinguishing NAC non-effectual group patients from the NAC effectual group. The absolute numbers of CD4^+^ and CD8^+^ TIL infiltration in tumors in the NAC non-effectual group were significantly lower than those in the effectual group. Similar findings were reported for the CD4^+^ T lymphocytes in the peripheral blood (*p’*s < 0.05). NAC effectual-related signature bacteria were proportional to these patients’ CD4^+^ T lymphocyte counts in peripheral blood and tumors (*p’*s < 0.05). CellMinor analysis showed that the CD4 mRNA expression level dramatically climbed with increased sensitivity of tumor cells to NAC drugs such as cyclophosphamide, cisplatin, and carboplatin (*p’*s < 0.05).

**Conclusions:**

The composition of the gut microbial community differs between BC patients for whom NAC is effective to those that are treatment resistant. The modulation of the gut microbiota on host CD4^+^ T lymphocytes may be one critical mechanism underlying chemosensitivity and NAC pathologic response. Taken together, gut microbiota may serve as a potential biomarker for NAC response, which sheds light on novel intervention targets in the treatment of NAC non-effectual BC patients.

## Introduction

Breast cancer (BC) has the highest incidence rate among all malignant tumors and is ranked the second cause of mortality in women (1). Patients with locally advanced breast cancer were often treated with neoadjuvant chemotherapy (NAC) (2), which is evident to reduce the primary tumor size in the breast prior to surgery to allow for breast conservation, thereby limiting the metastatic axillary lymph node, and increasing the surgical resection rate. However, some patients with breast cancer respond poorly to chemotherapy, the adoption of which not only pose little benefits but also can lead to potential chemotherapy toxicity, side effects, and disease progression in such population. Therefore, explorations in the influencing factors on the efficacy of NAC in such patients, in order to propose a predictive model for the patients’ responses to NAC, will be practically beneficial for planning better differential treatments for breast cancer.

Recent findings revealed that BC is related to microbial dysbiosis in both the gut microenvironment and breast tissue (3, 4). Multiple previous studies also reported that gut microbial composition modulates the chemotherapy efficacy and toxicity through key mechanisms including regulation of the translocation, steroid-hormone metabolism, and immune response to NAC drugs (5–8). More recently, it was reported that NAC could modulate the microbiome in the breast tumor tissue, as well as specific microbes, which are associated with tumor relapses through breast cancer signaling (9). Evidence from these human, animal, and *in vitro* studies together suggested that gut microbiota can be a promising biomarker for predicting the therapeutic outcome such as NAC efficacy in BC. Therefore, the multidimensional role of gut microbiota on cancer and treatment progress implies that gut microbiota can be a target for the development of personalized cancer therapeutics.

Much evidence has highlighted the prognostic value of tumor immune landscape and its role as therapeutic targets. The differential tumor immune landscape may contribute to the variations in clinical outcomes (10). Tumor infiltrating lymphocytes (TILs), commonly recognized as an immunological parameter and a morphological manifestation of anticancer immune response, represent a major infiltrating immune cell subpopulation, which consist of CD3^+^, CD4^+^, and CD8^+^ TILs. Much research has shown that TILs, which are known for their antitumor activities, also play a key role in modulating responses to NAC, with significant predictive values for the prognosis across breast cancer subtypes (11). For example, one study that assessed 3,771 BC patients reported that triple negative breast cancers (TNBCs), as well as HER2-positive (HER2^+^) breast cancer, were found with a significant increase in disease-free survival when TIL concentrations showed 10% escalation (12). However, the exact mechanism through which TILs regulate the responses to NAC in BC patients is still not clear.

Specific TILs are involved in the regulation of the microbiota, including CD3^+^, CD4^+^, and CD8^+^ TILs (13). Their functions range from providing help for regulation of intestinal barrier function, immunity, and metabolism to avoiding systemic and chronic inflammation, which have been proposed to underlie the impact of gut microbiota on carcinogenesis (14). For example, *Lactobacillus acidophilus* was reported to be able to modulate the immune response against breast cancer in murine models (15). *Bifidobacteria* also modify the induction of tumor−specific T cell and promote the entrances of circulating T cells into the tumor microenvironment in patients who received immunomodulator treatment (16). It is evidenced that lymphocytes-associated immune responses may affect tumorigenesis in breast tissue (17). At the same time, lymphocytes are regulated by microbes (18). For example, *Sphingomonas* was reported to engage in the effector maturation of CD8+ T cells, which are the most important immune cells that inhibit the growth of breast tumor cells (19). Altogether, it is reasonable to hypothesize that microbiomes may modulate the BC patients’ response to NAC by regulating their TIL induction.

Although logically reasonable, the impact of microbiome composition on BC patients’ responses to NAC and the role of TILs during this progress have remained unstudied. In addition, most previous studies focused on sequencing 16S rRNA or investigating biochemical interactions, which fail to comprehensively examine the entire taxonomies of the microbiota in BC. We thus conducted metagenomic analysis to explore the composition of gut microbiota and T lymphocyte subsets between NAC effectual patients and NAC non-effectual patients to fill such gaps. As in the literature, microbiota modulates the host response to chemotherapies, and immunity is implicated in the impact of microbiota on the development and progression of BC. By utilizing the machine-learning approach, this study also proposes a microbial signature for predicting the efficacy of NAC for BC and untangling the association between microbial signature, T lymphocyte subsets, as well as patients’ responses to NAC.

## Materials and Methods

### Patients

**Figure 1:**
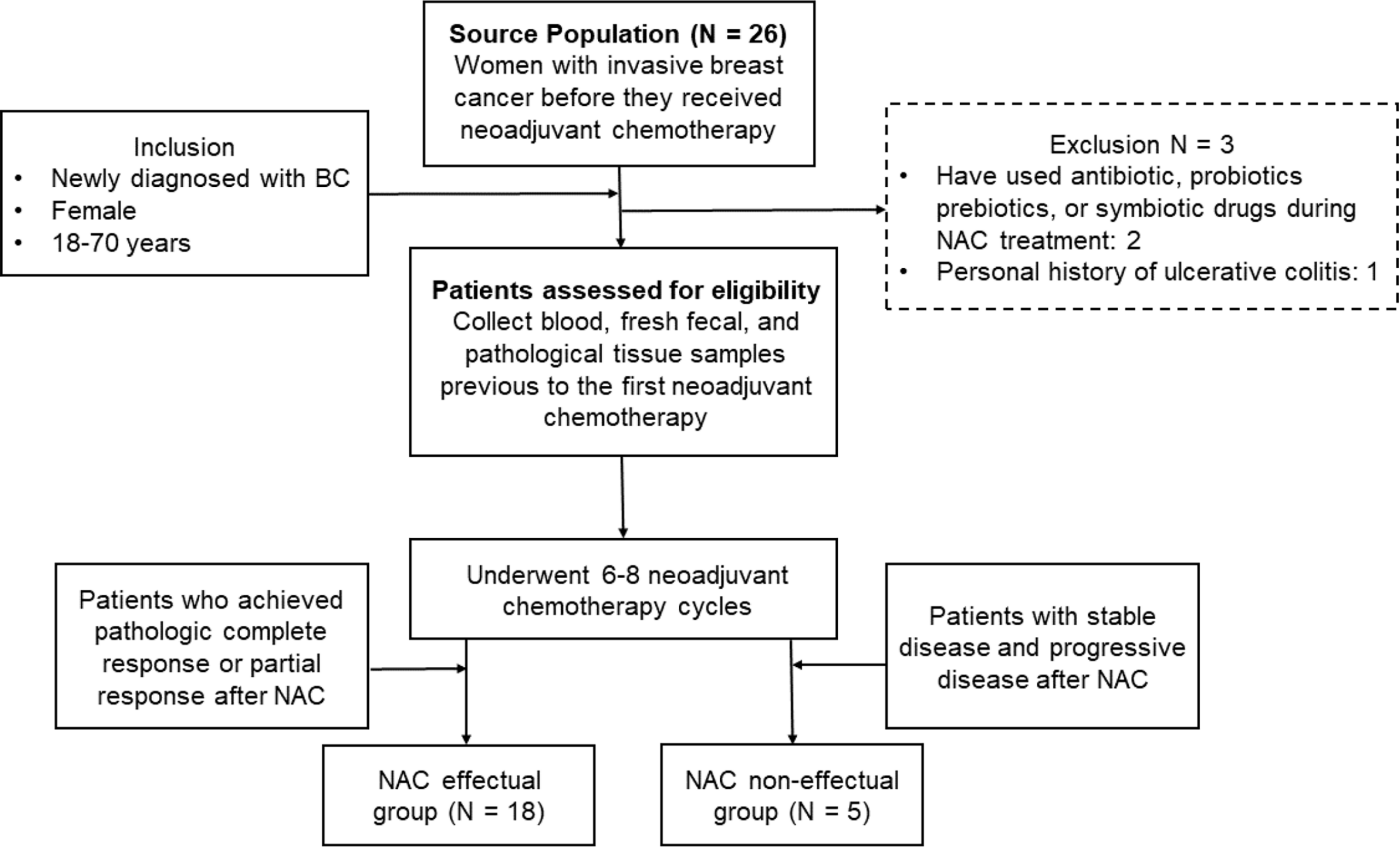
The recruitment of participants and the process of sample collection.

A total of 26 patients newly diagnosed with BC were recruited from the Sun Yat-Sen Memorial Hospital during half a year. Two of these 26 patients were excluded because they had used antibiotics, probiotics, prebiotics, or symbiotic drugs during NAC, and one was excluded because of a personal history of ulcerative colitis. All patients were female and between 18 and 70 years. Their clinical demographics (age, gender, BMI, menopause) and the histopathology of tumor (size, histologic grade, lymph node metastasis, the status of estrogen (ER), progesterone (PR), and human epidermal growth factor (HER2) receptors) were recorded along with the primary treatments they received (e.g., previous surgery, radiotherapy, or chemotherapy) (**Table 1**), since these indicators will have impacts on their responsiveness to NAC based on previous studies (20). The processes of participant recruitment and sample collection are depicted in **Figure 1**. To ensure the homogeneity of the patients in the different groups, detailed exclusion criteria included the following: (1) patients have received any chemotherapy, radiotherapy, endocrine therapy, or surgery prior to fecal sample collection; (2) patients who have used antibiotics, probiotics, prebiotics, or symbiotic drugs within 3 months prior to recruitment; (3) patients with concurrent malignant tumors; (4) patients with distant metastasis at initial presentation; (5) patients with comorbidities including severe heart, lung, liver, or kidney diseases; and (6) patients who were pregnant or lactating. Signed informed consents were obtained from all participants before sample collection. Ethic approval has been granted by the Ethics Committee of Sun Yat-Sen Memorial Hospital of Sun Yat-Sen University.

**Table 1:** Comparison of clinical indices and pathological data between NAC non-effectual group and NAC effectual group.

Characteristics		NAC non-effectual group n = 5	NAC effectual group n = 18	*p*-value
Age (years, mean ± SD)		52.80 ± 7.16	50.50 ± 10.41	0.58
BMI (kg/m^2^, mean ± SD)		21.99 ± 3.10	22.74 ± 3.20	0.65
Menopausal status	Premenopausal	2	10	0.64
	Postmenopausal	3	8	
Pathological Type (tumor size)	IDC	5	17	1.00
	LDC	0	1	
T stage	T1-T2	3	13	0.62
	T3-T4	2	5	
N stage	N0-N1	4	16	0.54
	N2-N3	1	2	
Histologic grade	Period II A-II B	3	11	1.00
	Period III A-III C	2	7	
ER expression	Positive	4	12	1.00
	Negative	1	6	
PR expression	Positive	3	6	0.34
	Negative	2	12	
HER2 expression	0-2+	5	10	0.12
	3+	0	8	
Ki-67 expression	≥20%	3	13	0.62
	<20%	2	5	
Blood TILs ratio (%, mean ± SD)				
CD3^+^TILs		69.34 ± 9.25	73.78 ± 8.63	0.23
CD4^+^TILs		**36.12 ± 3.95**	**41.39 ± 6.94**	**0.04**
CD8^+^TILs		23.92 ± 3.39	24.60 ± 6.37	1.00
CD4^+^/CD8^+^TILs		1.54 ± 0.35	1.77 ± 0.42	0.22
Therapy				
Cyclophosphamide-containing chemotherapy regimen	Yes	5	18	/
	No	0	0	
Anthracycline-containing chemotherapy	Yes	3	12	1.000
	No	2	6	
Taxol-containing chemotherapy	Yes	4	14	1.000
	No	1	4	
Herceptin targeted therapy	Yes	5	10	0.12
	No	0	8	
Chemotherapy cycle	≤6 cycles	4	14	1.000
	>6 cycles	1	4	
Chemotherapy interval	Intensive chemotherapy	1	3	1.000
	Conventional chemotherapy	4	15	

IDC, invasive ductal carcinoma; LDC, invasive lobular carcinoma; TILs, tumor infiltrating lymphocytes.

Tumors were categorized as ER, PR, HER2, and Ki-67 based on immunohistochemical testing results. If HER2 immunohistochemical testing result is 2+, two-probe method FISH is required. Bolded values indicate the significant results.

Blood and fresh fecal samples were collected from the patients the day before the first neoadjuvant chemotherapy. Reserved on ice, they were guaranteed to be transported within 2 h and immediately stored at −80°C until usage.

### Metagenomic DNA Sequencing and Annotation

Bacteria was investigated in 200 mg feces (7), and bacterial DNA was extracted with the stool sample DNA extraction kit (Guangzhou meiji biotechnology co., Ltd, China) following the manufacturer’s directions. We used the PE150 assay to sequence all the samples on the Illumina Hiseq 3000 platform. After removing contaminant distractions and the readings from human DNA (based on alignment with the SOAPdenovo aligner) in the raw data, clean reads for subsequent analysis were obtained. The qualified reads from each sample were aligned using SPAdes-v3.10.1 to satisfy the taxonomic assignments. By comparing the DIAMOND gene set with the NR database, we aligned our data to the NR database and completed the profile of taxonomic relative abundance. The estimation of its abundance was performed *via* evaluating the accumulation of all relative genes belonging to this feature.

### Blood Sample Analysis

Blood was collected from the patients prior to treatment for BC. Flow cytometry was used to determine T lymphocyte subset ratios in peripheral blood (21). For the detection of peripheral blood T lymphocyte subsets, the following antibodies were used: CD3-FITC/CD8-PE/CD4-APC (all from Becton-Dickinson). Cytofluorimetric analysis was performed with CytomicsTM FC500 (Beckman Coulter). CXP Cytometer and FlowJo Software (Tree Star Inc.) were used to analyze flow cytometry data. T lymphocyte subsets were identified as follows: CD3^+^ T cells, CD4^+^ T cells, and CD8^+^ T cells.

### Tissue Samples and TIL Scoring

All tissue samples were obtained from participants using core needle biopsies before the start of their NAC treatment. At the end of the clinical trial, TIL analysis was done by retrospectively reviewing the medical records. Clinicopathologic information was obtained using hematoxylin and eosin (H&E) stained slides and immunohistochemical slides for the standard biomarkers. Primary antibodies (Cell Signaling Technology, USA) specific for CD4 (1:400) and CD8 (1:400) were utilized for slide incubation overnight at 4°C. The secondary antibody (Cell Signaling Technology, USA) was incubated for 30 min at 37°C the following day, and the immunodetection was performed using a DAB kit (Beijing ComWin Biotech, China) following the manufacturer’s instructions. Absolute numbers of CD4^+^, CD8^+^ T cells, and their ratios were calculated in both intratumoral and stromal areas under 400× magnification using 3DHISTECH’s SlideViewer version 2.5 (3DHISTECH Ltd. Budapest, Hungary). A pathologist blinded to the purpose of our study independently examined and scored all the slides.

### Neoadjuvant Chemotherapy for Breast Cancer and Surgical Treatment

We employed the anthracycline-based regimen to all patients undergoing NAC. A total of 10 patients received the TEC regimens (Taxol 75 mg/m^2^+ Epirubicin 75 mg/m^2^ + Cyclophosphamide 500 mg/m^2^); 8 patients received the TC regimens (e.g., Cyclophosphamide 500 mg/m^2^ + Taxol 75 mg/m^2^); 3 patients received the EC regimens (e.g., Epirubicin 90 mg/m^2^ + Cyclophosphamide 600 mg/m^2^); 2 patients received the CEF regimens (e.g. Cyclophosphamide 500 mg/m^2^+ Epirubicin 75 mg/m^2^+ 5-Fluorouracil 500 mg/m^2^). The HER2^+^ BC patients received trastuzumab (Herceptin) triweekly for 12 months. Breast ultrasonography and breast MRI were used to assess the patients’ response to NAC treatment every 2 chemotherapy cycles. If tumor remission was detected, the NAC treatment would continue, and the patients would undergo breast surgery after 6–8 chemotherapy cycles. Meanwhile, under circumstances in which exacerbation or stable severity of disease was found, an alternative treatment plan with the chemotherapy regimen or performing breast surgery would be adopted. According to the Response Evaluation Criteria in Solid Tumors (RECIST 1.1), we categorized the subjects into two groups based on the treatment responses for tumor: the patients who achieved pathologic complete response (pCR) or partial response (PR) after NAC were assigned into the NAC effectual group, while those with stable disease (SD) and progressive disease (PD) were assigned into the NAC non-effectual group.

### Using CellMiner for System Analysis of the Relationship Between CD4 mRNA, CD8 mRNA Expression, and Reaction of Tumor Cells to NAC Drugs

CellMiner Cross-Database (CellMinerCDB, http://discover.nci.nih.gov/cellminer/) is a web-based application that provides both molecular and pharmacological data, and the tools to analyze these data within and across cancer cell line datasets from the National Cancer Institute (NCI), Broad Institute, Sanger/MGH, and MD Anderson Cancer Center (MDACC), which also allows systems pharmacology analysis of the largest publicly available database of anticancer drug activity (22). To understand the role of tumor immune landscape in the chemosensitivity and pathologic responses to NAC from BC patients, CellMinor (version 2.6) was used to analyze the relationship between the CD4 mRNA and CD8mRNA expression level and NAC drugs across cancer cell lines. The NAC drugs included in this analysis were anthracyclines (Doxorubicin, Epirubicin), cyclophosphamide, taxol derivative (Taxol, Docetaxel), platinum (Cisplatin, Carboplatin), Fluorouracil, alpha Methotrexate, Gemcitabine, Vinorelbine, Eribulin, and Ixabepilone.

### Statistical Analysis and Bioinformatics

Between-group comparison of clinical indices and pathological data was conducted with Student’s t-test, the chi-square test, and Mann–Whitney test using the SPSS 24.0 software (IBM, Inc., Chicago, IL). R software 3.6.1 was used to perform other analyses. Significant differences in alpha diversity based on the Chao estimate, Shannon, and Simpson index were measured using Wilcoxon rank-sum test by utilizing the “picante” package in R. Beta diversity was estimated by principal coordinate analysis (PCoA) of unweighted UniFrac analysis. Significant differences in abundance of genera between two groups were identified by linear discriminant analysis effect size (LEfSe) analysis. For LEfSe, the nonparametric factorial Kruskal–Wallis sum-rank test was employed to identify features with significant differential abundance, the effect size of which was calculated by subsequent linear discriminant analysis (LDA) (23). To detect the key signature microbiota at genus and species levels, we trained and run a random forest model (v.4.6–14 package in R 3.6.1) together with the 5-fold cross-validation to detect importance scores (mean decrease accuracy, MDA) and to examine the biomarkers’ importance rank ordering. The case probability was calculated using this set of species and a receiver operating characteristic (ROC) curve within the pROC package in R. Correlation analysis between T lymphocyte subsets and the signature microbiota was conducted using Spearman’s rank-based correlation. Statistical significance was set at *p*<0.05.

## Results

### Clinicopathological Characteristics in BC Patients From NAC Non-Effectual Group and NAC Effectual Group

Data from a total of 23 patients with invasive breast cancer were included in the study. According to RECIST 1.1, 18 cases were assigned into the NAC effectual group (the chemotherapy efficacy rate was 78.3%), and 5 cases were in the NAC non-effectual group. The two groups were comparable in terms of age, BMI, menopausal status, pathological type, clinical staging, ER expression, PR expression, HER2 expression, and Ki-67 expression (*p* > 0.05). Detailed patient clinical pathology data and clinical information are shown in **Table 1**.

### Taxonomic Characterization of Gut Microbiota in BC Patients From NAC Non-Effectual Group and NAC Effectual Group

**Figure 2:**
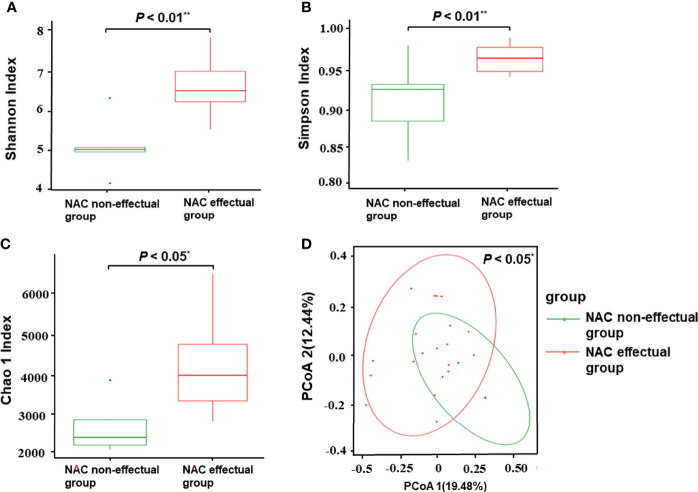
Comparisons of alpha-diversity and beta-diversity between the NAC effectual group (n = 5) and the NAC non-effectual group (n = 18). **(A–C)** Alpha-diversity of the two groups at the species level, measured in terms of the Shannon **(A)**, Simpson **(B)**, and Chao1 index **(C)**. **(D)** PCoA of unweighted UniFrac analysis showed that the overall fecal microbiota composition was different between the NAC non-effectual group and the NAC effectual group. NAC, neoadjuvant chemotherapy; PCoA, principal coordinate analysis. Superscript symbols indicate statistically significant differences between the two groups: *p < 0.05, **p < 0.01.

Considering that treatment factors may bias the predictive effect of the microbiota, we then used baseline specimens to explore the potential roles of discriminatory bacterial taxa as biomarkers. Based on the species profile, various alpha-diversity indexes (i.e., Chao1, Shannon, and Simpson index) were used to estimate gut microbiota richness and evenness in the sample. Compared with the NAC non-effectual group, the NAC effectual group exhibited highly diversified intra-individual characters, as indicated by the Chao 1 (p = 0.012), Shannon (*p* = 0.002), and Simpson (*p* = 0.005) index (Wilcoxon rank-sum test, **Figures 2A–C**).

Results of principal coordinate analysis based on unweighted UniFrac distance demonstrated significant differences in between-sample variability (beta-diversity) of the overall microbial composition between the NAC non-effectual group and the NAC effectual group (*p* = 0.046) (**Figure 2D**).

**Figure 3:**
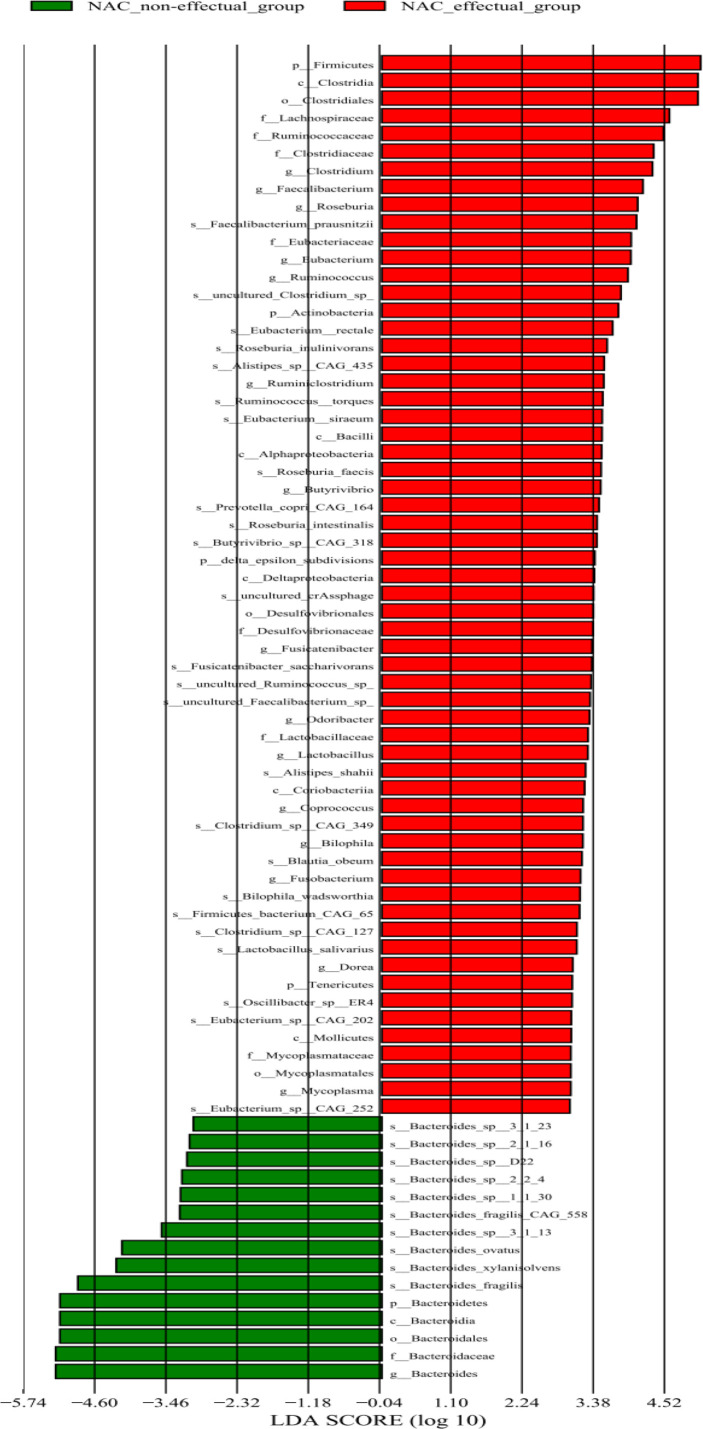
Relative abundance of 75 species differing significantly between the NAC non-effectual group and the NAC effectual group (LDA effect size analysis).

To further identify microbial signatures, defined by groups of bacterial taxa that were able to distinguish the NAC non-effectual group from the NAC effectual group patients, we performed LEfSe analysis on the fecal microbiota composition between the two groups. The entire abundance of genes determined the relative enrichments of gut microbiota. At the genus level, enriched levels of *Bacteroides* were found in the NAC non-effectual group patients, while 15 genera were found enriched in the NAC effectual group patients (LDA score >2.2, *p* < 0.05), 11 of which belonged to the phylum *Firmicutes* (*Clostridium, Faecalibacterium, Roseburia, Eubacterium, Ruminococcus*, *Ruminiclostridium, Butyrivibrio, Fusicatenibacter, Lactobacillus, Coprococcus, and Dorea* genera), followed by *Bacteroidetes* bacterial taxa (Odoribacter), and *Fusobacter* (*Fusobacterium* genus), *Proteobacteria* (*Bilophila* genus), and *Mycoplasma* genera. From the view of species, 25 microbes showed enrichments in the NAC effectual group, while the abundance of 10 species belonging to the *Bacteroides* genus was increased in the NAC non-effectual group (**Figure 3**). Hence, our analysis revealed that there are significant intergroup differences between NAC non-effectual patients and the NAC effectual group in their gut microbial composition.

### Gut Microbiome-Based Signature Discriminated the Therapeutic Response to NAC in BC Patients

**Figure 4:**
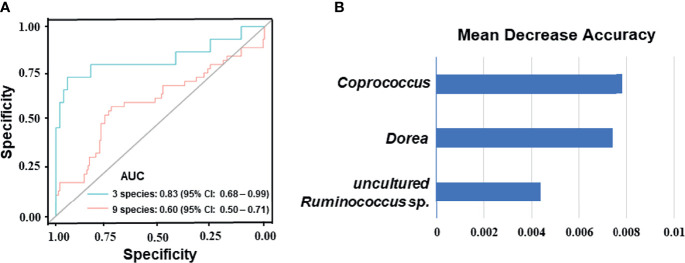
Disease classification based on gut microbiome signature. **(A)** Classification performance of the random forest model using the relative abundance of NAC- efficacy associated genera was assessed by the area under the ROC in BC patients. The combination of 3 signature bacteria: *Coprococcus, Dorea*, and uncultured *Ruminococcus* sp. The combination of 9 optimal species markers: *Bacteroides, Coprococcus, Dorea, Fusicatenibacter, Ruminococcus, Butyrivibrio* sp. CAG 318*, Lactobacillus salivarius, Bacteroides xylanisolvens*, and uncultured *Ruminococcus* sp. **(B)** Identification of the signature gut microbiota associated with NAC- efficacy by random forest. Fivefold cross-validation together with random forest was performed to determine the signature biomarkers. Detailed signature biomarkers’ random seed from the random forest is presented between the NAC non-effectual group and the NAC effectual group.

To identify signature bacteria that could predict the BC patients’ response to NAC, a random forest model with 5-fold cross-validation was performed to build a classification model with a training set consisting of 5 NAC non-effectual group patients and 18 NAC effectual group patients based on the above-mentioned 56 genera. Based on the mean decrease accuracy (**Figure 4B**), which depicts the ranked importance of signature microbial in differences between the NAC non-effectual group from the NAC effectual group patients, 9 optimal species markers were selected, including *Bacteroides, Coprococcus, Dorea, Fusicatenibacter, Ruminococcus, Butyrivibrio* sp. CAG 318*, Lactobacillus salivarius, Bacteroides xylanisolvens*, and uncultured *Ruminococcus* sp. We found that the combination of 3 signature bacteria (*Coprococcus, Dorea*, and uncultured *Ruminococcus* sp.) worked best to distinguish the NAC non-effectual group from the NAC effectual group patients with an AUC = 0.833 (95% CI: 0.678–0.989). However, employing all the 9 genera (AUC: 0.604, 95% CI: 0.499–0.71) did not significantly improve the predictive performance (**Figure 4A**).

### Analysis of Tumor-Infiltrating Lymphocytes in Peripheral Blood and Tumor Tissue of BC Patients Treated by NAC

As shown in **Table 1**, after controlling for age, BMI, and menopausal status, results of the Mann–Whitney test showed that, although the NAC effectual group had increased levels of the peripheral blood T lymphocyte subset ratio than the NAC non-effectual group, the difference was only significant in CD4^+^T lymphocyte between the two groups (*p* < 0.05).

**Figure 5:**
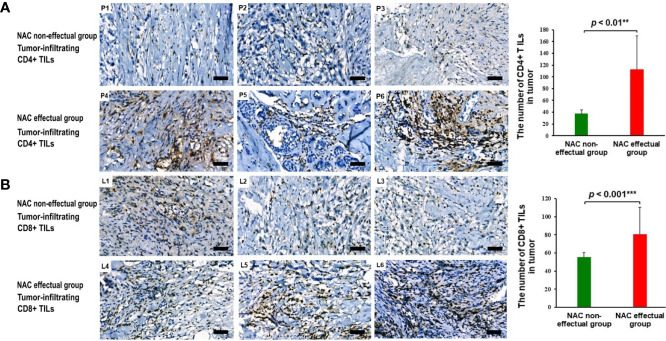
**(A)** The presence and abundance of tumor-infiltrating CD4^+^ TILs as assessed with IHC staining between representing NAC non-effectual patients (P1, P2, P3) and NAC effectual patients (P4, P5, P6). Scale bars, 50 μm. **(B)** The presence and abundance of tumor-infiltrating CD8^+^ TILs as assessed with IHC staining between representing NAC non-effectual patients (L1, L2, L3) and NAC effectual patients (L4, L5, L6). Scale bars, 50 μm. IHC, immunohistochemistry. Data show means ± s.d. ** denotes *p* < 0.01 by Student’s *t*-test. *** denotes *p* < 0.001 by Student’s *t* test.

As shown in **Figure 5**, infiltration of CD4^+^ and CD8^+^TILs was found in the intratumoral and stromal areas; infiltration of CD4^+^ and CD8^+^ TILs was significantly higher in the tumors of the NAC effectual group. After controlling for age, BMI, and menopausal status, results of the Mann–Whitney test showed that in the NAC effectual group, the absolute numbers of CD4^+^ and CD8^+^ TIL infiltration were significantly higher than those in the NAC non-effectual group (*p* < 0.001, *p* < 0.01, respectively).

### Signature Bacteria Associated With T Lymphocyte Cell Subsets in BC Patients Treated by NAC

**Figure 6:**
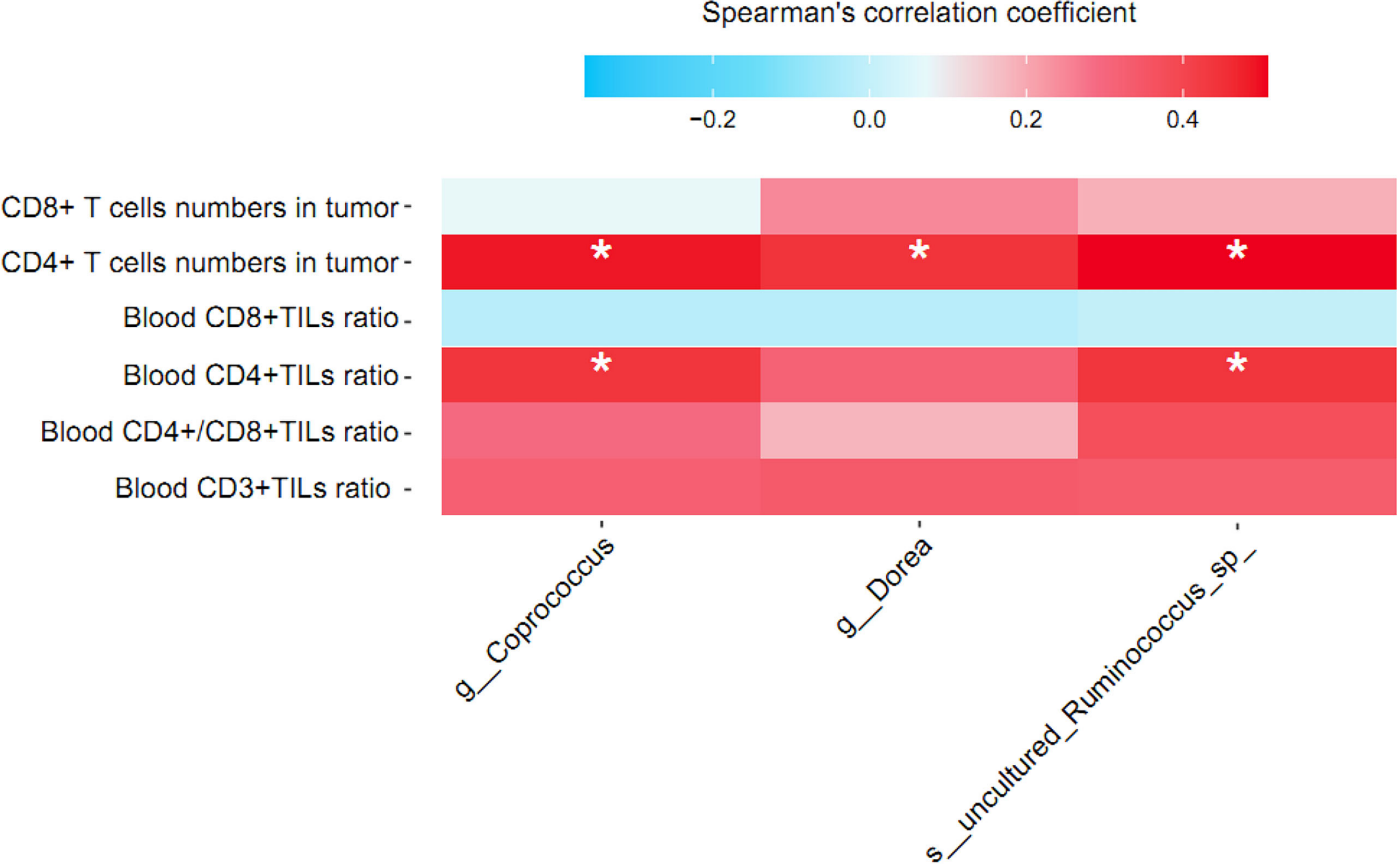
Correlation between relative abundance of signature gut microbiota and T lymphocyte cell subsets in breast cancer patients treated by NAC. Partial Spearman’s rank correlation coefficient is indicated using a color gradient: red indicates positive correlation; blue, negative correlation. TIL, tumor infiltrating lymphocyte. * denotes *p* < 0.05.

We used partial Spearman’s rank-based correlation tests (controlling for age, BMI, and menopausal status) to uncover the associations between blood and tumor-infiltrating T lymphocytes and NAC effectual-related signature bacteria in BC patients treated by NAC. Two signature bacteria *Coprococcus* and uncultured *Ruminococcus* sp. were found to be significantly overrepresented in patients with advanced blood CD4^+^ TIL counts compared to those with lower blood CD4^+^ TIL counts (*r* = 0.44 and 0.44, respectively, all *p’*s<0.05). All the three signature bacteria *Coprococcus, Dorea*, and uncultured *Ruminococcus* sp. were significantly and positively correlated with CD4^+^ T lymphocyte in tumor (r = 0.49, 0.44, and 0.51, respectively, all *p’*s<0.05). However, no significant correlation between CD3^+^ and CD8^+^ T lymphocyte and signature bacteria was found (**Figure 6**).

### Correlations Between CD4, CD8 mRNA Expression Level, and the Sensitivity of Tumor Cells to NAC Drugs

**Figure 7:**
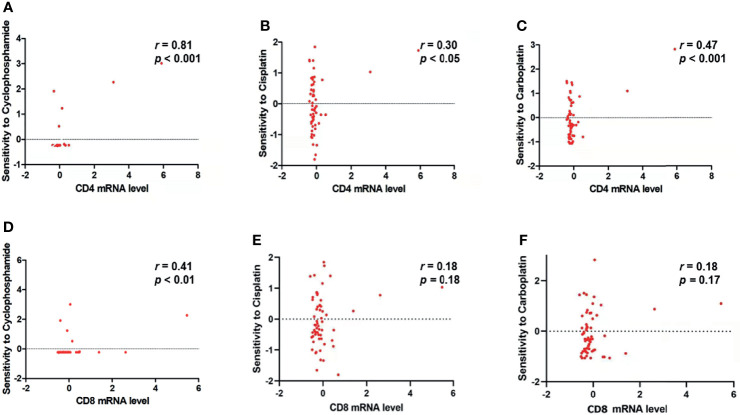
CD4 mRNA and CD8 mRNA expression level is related to the sensitivity of tumor cells to NAC drugs. **(A–C)** CD4 mRNA expression level is significantly and positively correlated with the sensitivity of tumor cells to **(A)** cyclophosphamide (*r* = 0.81, *p* < 0.0001), **(B)** cisplatin (*r* = 0.30, *p* < 0.05), and **(C)** carboplatin (*r* = 0.47, *p* < 0.001). **(D)** CD8 mRNA expression level is significantly and positively correlated with the sensitivity of tumor cells to cyclophosphamide (*r* = 0.41, *p* < 0.01). **(E, F)** CD8 mRNA expression level is not significantly correlated with the sensitivity of tumor cells to **(E)** cisplatin (*r* = 0.18, *p* = 0.18) and **(F)** carboplatin (*r* = 0.18, *p* = 0.17).

As shown in **Figure 7**, the result of CellMiner Cross-Database analysis showed that a higher level of CD4 mRNA expression was significantly associated with higher sensitivity of cancer cell lines to cyclophosphamide (*r* = 0.81, *p*< 0.001, **Figure 7A**), cisplatin (*r* = 0.30, *p*< 0.05, **Figure 7B**), and carboplatin (*r* = 0.47, *p*< 0.001, **Figure 7C**). As for CD8 mRNA expression, it was only found significantly and positively correlated with cyclophosphamide (*r* = 0.41, *p*< 0.01, **Figure 7D**) but not with cisplatin (*r* = 0.18, p = 0.18, **Figure 7E**) or carboplatin (*r* = 0.18, *p* = 0.17, **Figure 7F**).

## Discussion

NAC has been routinely prescribed for patients with locally advanced breast cancer, which can relieve local tumor burden and create favorable conditions for surgery. However, not all breast cancer patients can benefit significantly from NAC. Therefore, it is of great clinical significance to identify effective predictive markers for BC patients’ responses to NAC treatment. There is growing evidence that altered gut microbiome correlates with the development of multiple tumor types and modulates the host response to chemotherapeutic drugs. In this study, we performed a comprehensive metagenomic comparison of gut microbiota in highly homogenous breast cancer patients treated by NAC. We also investigated the association between gut microbiome and BC patients’ responses to NAC treatment, as well as the related mechanism. The results highlighted the characteristics of the NAC effectual group defined as abundant bacterial diversity and variation in microbial community. Given the necessity of predicting BC patients’ responses to NAC treatment, a model with discriminatory diagnostic power was exploratorily trained. The most notable finding was that 3 signature bacteria were associated with TIL concentrations and NAC response in BC patients.

The fecal microbiome in patients in the NAC non-effectual group showed decreased species richness and distinct within-sample diversity compared with patients in the NAC effectual group. Researchers had reached consensus that low bacterial diversity, as one of the major types of gut dysbiosis, was involved in a variety of diseases (4). The Goedert team published a series of case-control studies on the relationship between gut microbiota and breast cancer, which indicated that BC patients had decreased α and β diversity of gut microbiota compared to healthy controls, and they also had gut microbiome dysbiosis that was characterized by an increase in the abundance of *Clostridium* (24–26). Moreover, highly diverse fecal microbiome endorsed significantly longer progression-free survival in contrast to low or moderate diversities of microbiota (4). These studies, together with the current findings, imply that lowered bacterial diversity may increase the possibility of resistance to NAC treatment and a lowered NAC efficacy, which can thus be an important predictive index.

Regarding relative abundances in microbiota composition, the NAC non-effectual group and the NAC effectual group showed differences in the abundance of 56 bacteria species. The levels of the major component of the adult fecal microbiota, phylum Firmicutes (including *Clostridium, Faecalibacterium, Roseburia, Eubacterium, Ruminococcus*, *Ruminiclostridium, Butyrivibrio, Fusicatenibacter, Lactobacillus, Coprococcus*, and *Dorea* genera), were decreased while *Bacteroides*, that of the core genus of the phylum Bacteroidetes, were increased in the NAC non-effectual group relative to the NAC effectual group. The pro-inflammatory bacteria *Bacteroides* showed the most effective association with the NAC non-effectual group patients, which is in line with the previous findings that *Bacteroides* were positively associated with breast tumors and the severity of cancer (8, 27). In addition, a significant decrease in the Firmicutes phylum and an increase in the Bacteroidetes phylum were reported to be indicative of poorer cancer outcomes. Interestingly, an increased relative abundance of *Bacteroides* was also reported, which might cause increased intestinal barrier permeability and inflammation in women diagnosed with invasive breast cancer, which might influence their fear for cancer recurrence (28). Further study compared the differences in gut microbiota between before and after chemotherapy and found that chemotherapy induced gastrointestinal mucositis and gastrointestinal reaction, which was associated with severe gut microbiome dysbiosis such as a decrease in Firmicutes (29). Remarkably, in the NAC non-effectual group, we also identified a decrease in antioxidant indolepropionic acid producers (e.g., *Clostridium, Fusobacterium, Fusicatenibacter*), which could maintain or promote intestinal permeability and systemic immunity (30). While no study, to date, has documented the impact of microbiota populations on the NAC response in breast cancer, our results, as well as the aforementioned findings, indicate that BC patients’ microbial signatures such as a decreased ratio of Firmicutes/Bacteroidetes may influence their therapeutic outcome. In addition, the microbial community in the NAC non-effectual group patients may shift toward the depletion of butyrate-producing and indolepropionic acid-producing bacteria, which may modulate the activity and efficacy of NAC treatment for them.

Our results from the random forest model found that three bacteria, *Coprococcus, Dorea*, and uncultured *Ruminococcus* sp., were most useful for distinguishing the NAC non-effectual group patients from the NAC effectual group patients. The accumulation of numerous metabolites from human gut microbiota rendered systemic influences on the host. An increase in butyrate-producing bacteria (e.g., *Coprococcus* and uncultured *Ruminococcus* sp.) was observed in the NAC effectual group patients. In addition, *Dorea*, which was found to be an acetate and lactate producer, and may serve as a substrate for butyrate production, was also overrepresented in NAC responders. Besides its anti-inflammatory roles, butyrate also works to immune cells *via* specific G-protein-coupled receptors expressed on the surface (31). In addition, the heightened levels of these butyrate-producing bacteria promote the production of short-chain fatty acids (SCFAs) and contribute to a more favoring microbial profile (31). Increased production of SCFA by microbiota was suggested to pose health benefits through anti-inflammatory effects (23) and to play an important role in protecting the intestinal barrier function and ameliorating mucosal inflammation (28, 32). Interestingly, higher abundance of butyrate-producing bacteria was also significantly associated with a better response to immunotherapy (33–35). However, consistent with some of the findings from immunotherapy and chemotherapy research, no direct association between enrichment of the butyrate-producing microbes in responders and treatment responses was found in our study. Fortunately, published experimental studies have provided some mechanistic insights into this issue.

CD4^+^ and CD8^+^ T cells as immunological parameters were well known to assist the activation of the antigen-presenting cells *via* cytokine secretion, which favored the prognosis of breast cancer (10, 36). Within the result of the CellMiner Cross-Database for system analysis of the relationship between NAC drugs and CD4 mRNA expression across cancer cell lines, we found that the CD4 mRNA expression level was significantly associated with the sensitivity of tumor cells to NAC drugs such as cyclophosphamide, cisplatin, and carboplatin. Moreover, the CD8 mRNA expression level was also significantly and positively correlated with the sensitivity of tumor cells to cyclophosphamide. This is in line with past studies (36) and also correlated evidence that CD4+ and CD8+ TILs may serve as biomarkers for the clinical outcome of NAC in BC patients.

Substantial data supported that the regulations of the immune system by composed gut microbiota could lead to huge impacts to the efficacy and toxicity of antitumor therapy (37, 38). Nevertheless, the underlying physiological mechanisms in the antitumor-related immune response remained unclear. Research suggested that gut microbiota may participate in the plasticity of CD4+ T cells in the tumor microenvironment and cause antitumor or tumor-promoting immune responses, thereby exerting anticancer or tumor-promoting effects (39), but the specific signature bacteria that related to the immune function in the breast cancer patients are rarely reported. In this study, we found that the NAC effectual group showed a higher level of peripheral blood CD4^+^T cells and higher absolute numbers of CD4^+^ and CD8^+^ TIL infiltration in tumor tissues than the NAC non-effectual group, which suggest that the NAC effectual group patients may have better immune functions compared with the NAC non-effectual group. Likewise, we also found that a higher abundance of *Coprococcus, Dorea*, and uncultured *Ruminococcus* sp. may contribute to the higher levels of peripheral blood and tumor-infiltrating CD4^+^TILs in BC patients. These findings suggest that enhanced antigen presentation or CD4^+^T cell recruitment in the local tumor environment by microbiota holds their momentousness in the NAC treatment responders. This is consistent with the previous studies (40, 41) and in support of our research hypothesis that gut microbiota may regulate the efficacy of the NAC *via* its interactions with immune cells.

Many studies have documented the beneficial role of *Coprococcus* in promoting healthy immune function, and decreased *Coprococcus* representation has been linked with several diseases including lung cancer (42), psoriatic arthritis (43), and immune-mediated inflammatory disease (44). As for uncultured *Ruminococcus* sp., it was found to have an anti-inflammatory effect (45) and was involved in immunomodulatory and promoting glucose homeostasis (46). Previous studies have also reported that the genus *Dorea* is positively correlated with the response to NAC in rectal cancer patients (47). However, there is no previous research that has examined the relationship between CD4^+^ T cells and gut microbiota in BC patients. Our findings echoed with one existing study conducted in patients with metastatic melanoma, where before receiving anti-PD-1 immunotherapy, patients who later responded to immunotherapy were found with more abundant baseline *Bifidobacterium* and *Enterococcus* in their gut microbiota (48). Taken together, these results provide suggestive evidence that *Coprococcus, Dorea*, and uncultured *Ruminococcus* sp. may be closely linked to TILs such as CD4^+^ T cells; therefore, modulating these three bacteria may further affect NAC treatment outcomes in BC patients by regulating their immune function. *Coprococcus, Dorea*, and uncultured *Ruminococcus* sp. may serve as novel bacterial biomarkers in predicting the activity and efficacy of NAC treatment in BC patients. This implication is worthy of further verification in a larger sample. In the future, developing a novel therapeutic approach using bacteriophages to target the specific signature bacteria identified to be related to NAC efficacy in this study, which will precisely edit the intestinal microbiota, should be a promising intervention strategy to alter the intestinal microbiomes of and improve the therapeutic effect in breast cancer patients.

The advantages of the current study mainly lie in the following areas: i) methodology, such as a collection of highly homogeneous samples prior to NAC treatment; ii) utilization of the metagenomic analyses; and iii) the adoption of database analysis such as CellMiner Cross-Database analysis. Yet, there are still limitations that need to be addressed. First, this study is preliminary. The sample size is small and therefore the findings should be considered with caution, and repetition of bacteria signature related to the response rate to NAC *in vivo* is desired to further establish the causal relationships between gut microbiota changes, immune activation, and neoadjuvant chemotherapy efficacy. Multicenter studies involving diverse populations across different ages and breast cancer types would also provide further definite evidence to ensure the predictive role of signature bacteria on NAC response. Second, despite strict criteria applied to the inclusion/exclusion of participants, they were recruited from the same region (i.e., similar dietary habits existed among the subjects), and patients’ dietary habits and lifestyles were not controlled, which might induce confounding bias to our results. Third, this study was not able to indicate the possible functional relevance of these microbes in the patients. The possible mechanisms such as immunoregulation, which may elucidate the relationships between signature bacteria, TILs, and NAC efficacy, were not examined in this study without measuring the serum inflammatory biomarkers and related microbial metabolomics, such as interleukin-6, interleukin-1 receptor antagonist, tumor necrosis factor-α, and the tryptophan’s metabolites (49). Future studies should adopt these inflammatory biomarkers as well as the microbial metabolite to examine these mechanisms. Fourth, this research is a cross-sectional study, which cannot provide the dynamics between gut microbiota and NAC efficacy. A prospective cohort trial to show the longitudinal changes in the NAC efficacy-associated microbiota is needed in the future.

## Conclusion

**Figure 8:**
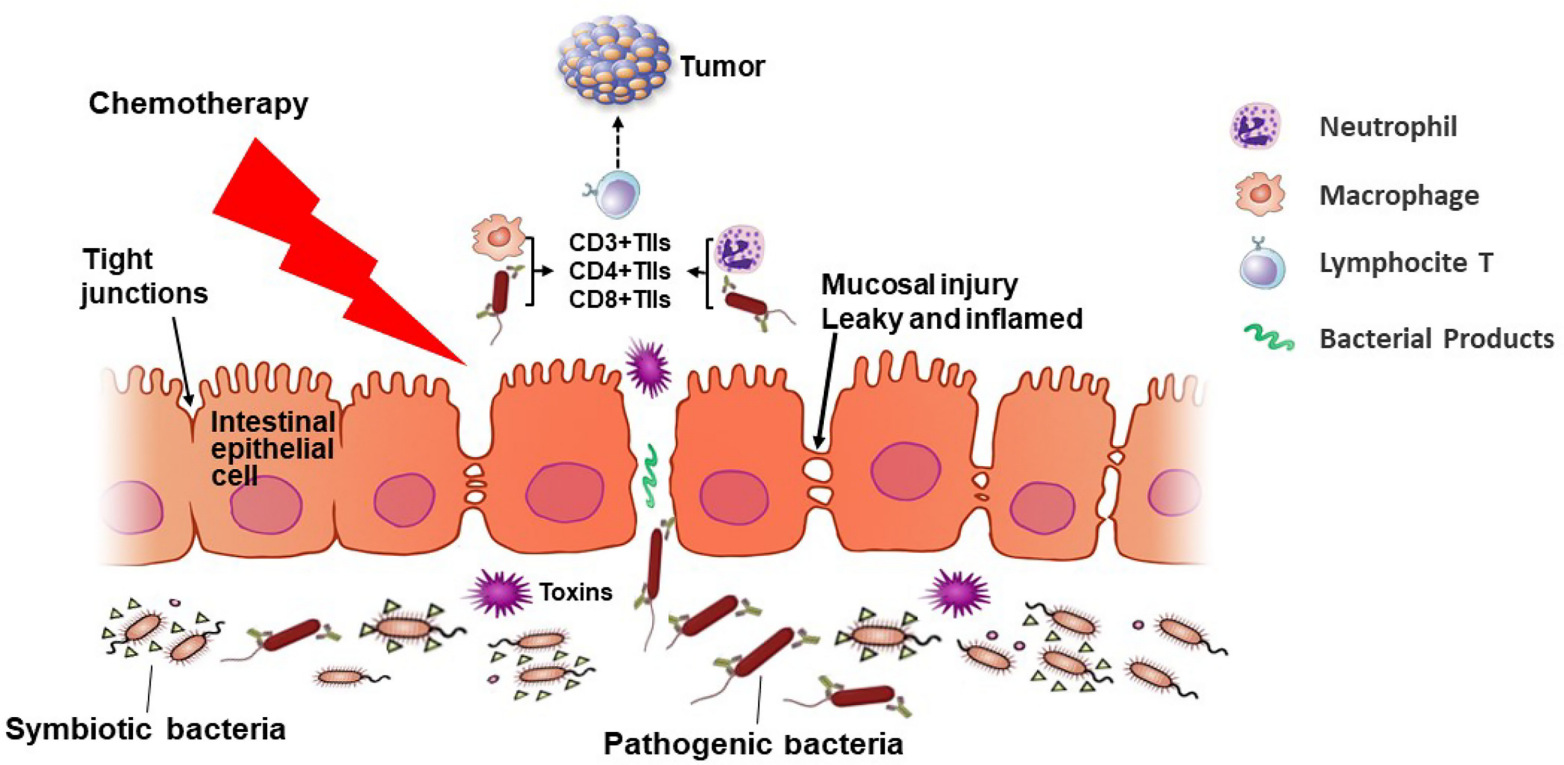
An overview of the microbiota–T lymphocyte (TILs) interactions that modulate neoadjuvant chemotherapy (NAC) efficacy. NAC treatment induces changes in the gut microbiota diversity, causing inflammation and damage to the mucosal barrier of cancer patients, permitting pathogenic bacteria to cross the intestinal barrier and enter lymphoid organs. Then the intestinal microbiota may mediate the induction of TILs such as CD3^+^, CD4^+^, and CD8^+^ cells in patients undergoing NAC. When the tight junctions between epithelial cells are broken and the intestinal permeability reduces, pathogenic bacteria interact with immune cells, regulating the response rate of the NAC treatment.

This study contributes to identifying the differential composition of the gut microbiota community between the NAC non-effectual group and NAC effectual group patients. We developed a prediction model for neoadjuvant chemotherapy response based on the relative abundance of gut microbiota. The bacteria signature related to the response rate to NAC and cancer outcome also links to TIL levels, especially CD4^+^ T cells (as **Figure 8** proposes). The findings raise the possibility of using novel microbiota biomarkers in the evaluation of the responsiveness to NAC treatment for BC patients and put forward new strategies for regulating gut microbiota as potential therapeutic targets. Future understanding about the possible role of microbiota, especially *Coprococcus, Dorea*, and uncultured *Ruminococcus* sp., and their interaction with TILs in improving breast cancer outcome is warranted.

## Data Availability Statement

The datasets presented in this study can be found in online repositories. The names of the repository/repositories and accession number(s) can be found below: Zenodo under accession number 10.5281/zenodo.5939855 (https://zenodo.org/record/5939855#.YfkzK5pBxPY).

## Ethics Statement

Permission has been granted by the Ethics Committee of Sun Yat-Sen Memorial Hospital of Sun Yat-Sen University. The patients/participants provided their written informed consent to participate in this study.

## Author Contributions

Each author contributed substantially to the paper. YL conceived the study hypothesis, performed data analysis, and drafted the manuscript. BD, WW, and JW had made contributions to the conception, design of the work, sample collection, and data analysis. HJ contributed to data analysis and validation. KC and KH contributed to reviewing the literature and sample collection. SH and YY conceived the study hypothesis, revised it critically for important intellectual content, and supervised the writing of the manuscript. All the authors read and approved the final manuscript.

## Funding

This work was supported by grants from the Fundamental Research Funds for the Central Universities (20ykjc03), the National Science Foundation of China (82071859, 81772837, 82071860, 82003176), Guangdong Natural Science Foundation (2018A0303130322), the Science and Technology Foundation of the Guangdong Province (2019A050510016), and Guangdong Innovation and Entrepreneurship Team Projects (2019BT02Y198).

## Conflict of Interest

The authors declare that the research was conducted in the absence of any commercial or financial relationships that could be construed as a potential conflict of interest.

## Publisher’s Note

All claims expressed in this article are solely those of the authors and do not necessarily represent those of their affiliated organizations, or those of the publisher, the editors and the reviewers. Any product that may be evaluated in this article, or claim that may be made by its manufacturer, is not guaranteed or endorsed by the publisher.
